# Parahiraciini (Hemiptera, Fulgoromorpha, Issidae): one new genus, two new species and three new subtribes

**DOI:** 10.3897/zookeys.997.52857

**Published:** 2020-11-25

**Authors:** Thierry Bourgoin, Menglin Wang

**Affiliations:** 1 Institut de Systématique, Évolution, Biodiversité, UMR 7205 MNHN-CNRS-Sorbonne Université-EPHE-Univ. Antilles, Muséum national d’Histoire naturelle, CP 50, 57 rue Cuvier, F-75005 Paris, France Muséum national d’Histoire naturelle Paris France; 2 Key Laboratory of Southwest China Wildlife Resources Conservation of the Ministry of Education, China West Normal University, Nanchong, Sichuan Province, 637009, China China West Normal University Nanchong China

**Keywords:** Auchenorrhyncha, Fulgoroidea, molecular, morphology, phylogeny, planthopper, taxonomy

## Abstract

A new genus *Pusulissus***gen. nov.** with two new species *P. phiaoacensis***sp. nov.** and *P. coronomensis***sp. nov.** are described respectively from Vietnam and China in the tribe Parahiraciini of the family Issidae. A molecular phylogeny using combined genes (18S, 28S, COX1 and Cytb) confirms its placement in the tribe Parahiraciini. The tribe is divided into three subtribes: Scantiniina**subtribe nov.** with the type genus *Scantinius* Stål, 1866, and Vindilisina**subtribe nov.** with type genus *Vindilis* Stål, 1870 plus *Nisoprincessa* Gnezdilov, 2017, and the nominal subtribe Parahiraciina Cheng & Yang, 1991 **subtribe nov.** The characteristics of these subtribes are provided, with a key to identification. Genus *Folifemurum* Che, Zhang & Wang, 2013 is transferred to HemisphaeriiniMongolianina, and genus *Gelastyrella* Yang, 1994 is maintained as a valid name.

## Introduction

The classification of the planthopper family Issidae Spinola, 1839 has been greatly modified in the recent years (reviewed in Wang et al. 2016). Emeljanov (1990) recognized seven subfamilies in the family Issidae Spinola, 1839 (including Acanaloniinae Amyot & Serville, 1843, Bladininae Kirkaldy, 1907, Caliscelinae Amyot & Serville, 1843, Tonginae Kirkaldy, 1907 and Trienopinae Fennah, 1954 which have now been dispatched to other planthopper families). In comparison Cheng and Yang (1991a, b) restricted the family Issidae to only five subfamilies: Issinae, Tonginae, Caliscelinae, Hemisphaeriinae Melichar, 1906 and an additional new monogeneric subfamily Parahiraciinae Cheng & Yang, 1991. This last taxon was based on the genus *Parahiracia* Ôuchi, 1940, originally described within the family Tropiduchidae before being transferred into Issidae by Fennah (1982). This distinction was not followed by Chan and Yang (1994: 86) and Emeljanov (1999) who transferred the genus to the issid Thioniini Melichar, 1906. Only ten years later, Gnezdilov (2003: 306) recognized again *Parahiracia*’s particular taxonomic position in a separate monospecific tribe Parahiraciini Cheng & Yang, 1991, based on its highly modified hindwing. This separation was confirmed later by molecular analyses (Wang et al. 2016).

A year after Parahiraciini’s rehabilitation, Gnezdilov et al. (2004: 221) synonymized *Parahiracia* under *Fortunia* Distant, 1909 and added *Scantinius* Stål, 1866, *Pterygoma* Melichar, 1903, *Prosonoma* Melichar, 1906 (synonymized later with *Bardunia* Stål, 1863) and *Bardunia* to this tribe. In addition to the long incision between the first two hindwing lobes, the authors characterized the tribe by ‘a strongly protruding metope in shape of probocis and well developed bi- or tri-lobed hindwings’. Another year later, Gnezdilov and Wilson (2005) added two more genera: *Pinocchias* Gnezdilov & Wilson, 2005, *Narinosus* Gnezdilov & Wilson, 2005 and synonymized *Clipeopsilus* Jacobi, 1944 with *Fortunia*. In 2007, Gnezdilov and Wilson transferred *Pterygoma* to Caliscelidae, but added *Mincopius* Distant, 1909 and *Flavina* Stål, 1861 to the tribe, with a key to the seven Parahiraciini genera (Gnezdilov and Wilson 2007). Zhang and Chen (2008, 2009, 2010, 2012) successively added five more genera: *Neodurium* Fennah, 1956, *Tetricodes* Fennah, 1956, *Fusiissus* Zhang & Chen, 2010, *Paratetricodes* Zhang & Chen, 2010, and *Neotetricodes* Zhang & Chen, 2012. In 2013, *Folifemurum* Che, Zhang & Wang, 2013 was described (Che et al. 2013). In his review of Issidae of the world, Gnezdilov (2013) added *Duriopsilla* Fennah, 1956, listing therefore 14 genera in the tribe.

Chen et al. (2014) re-established *Gelastyrella* Yang, 1994 as a valid taxon and one year later, Wang et al. (2015) and Meng et al. (2015) added respectively *Tetricodissus* Wang, Bourgoin & Zhang, 2015 and *Brevicopius* Meng, Qin & Wang, 2015 to the Parahiraciini. In the same year, Gnezdilov (2015) discussed the possibility that *Pseudochoutagus* Che, Zhang & Wang, 2011 and *Thabena* Stål, 1861 (including its synonyms *Cibyra* Stål, 1861, *Gelastyra* Kirkaldy, 1904, *Gelastyrella* Yang, 1994 (sic: not mentioning Chen et al. (2014)’s rehabilitation) and *Borbonissus* Bonfils, Attié & Reynaud, 2001) might belong to Parahiraciini. The transfer of these two genera as well as *Macrodarumoides* Che, Zhang & Wang, 2012, was formally confirmed by Wang et al. (2016), supported by molecular phylogeny analyses. However, they excluded *Folifemurum* moving it to Hemisphaeriini (Hemisphaeriina). The authors briefly characterized further Parahiraciini with the hindwing conformation: a deep narrowed incision of hindwing with a Pcu-A1 lobe distinctly wider than ScP-R-MP-Cu lobe, a short and thin A2 lobe in which A2 vein is often absent, free Pcu and A1, not partially fused. The same year, *Rhombissus* Gnezdilov & Hayashi, 2016 was described (Gnezdilov and Hayashi 2016). Finally, Gnezdilov (2017) added *Nisoprincessa* Gnezdilov, 2017 and *Thabenula* Gnezdilov, Soulier-Perkins & Bourgoin, 2011, and transferred the Philippines genus *Vindilis* Stål, 1870 into the tribe (Gnezdilov 2018).

Interestingly, Parahiraciini are also known from one fossil genus, *Bolbossus^†^* Gnezdilov & Bourgoin, 2016, from Baltic amber (dated from Priabonian: 37.8–33.9 Mya) (Gnezdilov and Bourgoin 2016).

**Figure 1.**
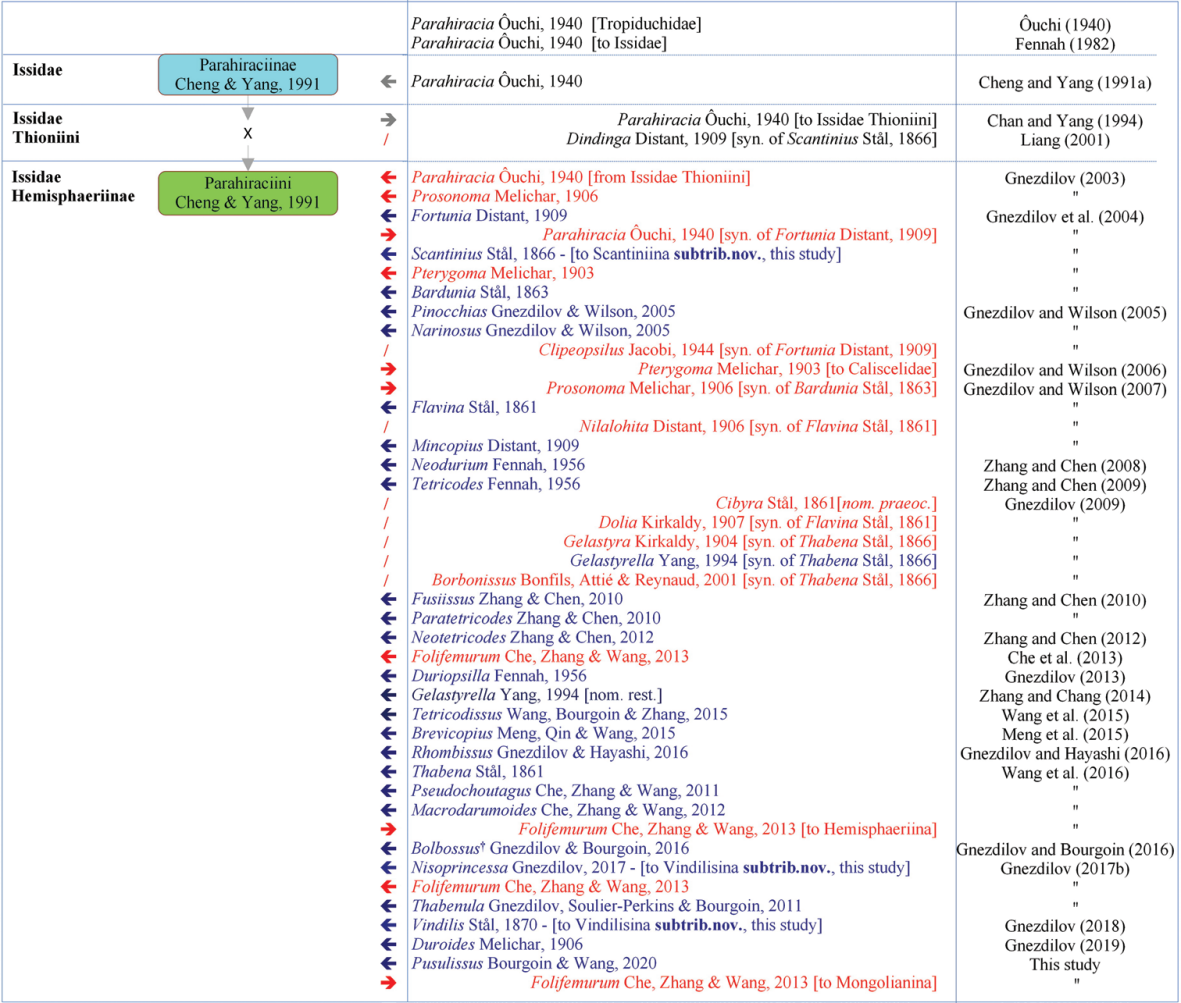
Chronological account of the genera in the tribe Parahiraciini Cheng & Yang, 1991. Box colors follows FLOW (Bourgoin 2020) standard colors with pale blue: taxon protonym and green: accepted taxon. Left arrows indicate when a genus was included in the tribe and right ones when excluded. Red and blue arrows refer respectively to taxa excluded or included in Parahiraciini.

In fact, in some 15 years since Parahiraciini was recognized again by Gnezdilov (2003), the tribe shows already a rather complex history with up to 26 genera with 82 species (Bourgoin 2020). In this study we retain 26 genera including the new one described. Figure 1 summarizes this already rich and complex chronological account of Parahiraciini generic taxa.

In addition to this synthetic taxonomic review, we also describe a new genus (already mentioned in Wang et al. (2016)’s molecular phylogeny under the name ‘Gen. nov. *apud Tetricodes*’) with two new species from Vietnam and China. A molecular phylogeny, using the combined genes (18S, 28S, COX1 and Cytb) of the already sequenced taxa, allows us to place this new genus into Parahiraciini and to briefly discuss this placement. Moreover, the large diversity of the taxa now included in the tribe also allows us to better clarify its morphological characteristics. Accordingly, the tribe is divided into three subtribes: Scantiniinia subtribe nov., Vindilisina subtribe nov. and the nominal subtribe Parahiraciina Cheng & Yang, 1991 subtribe nov., for which morphological characteristics are compared.

## Materials and methods

Morphological interpretations and subsequent terminologies follow Bourgoin (1987) and Bourgoin (1993) respectively for male and female genitalia and Bourgoin et al. (2015) for wing venation. The metatibiotarsal formula provides the number of spines on the side of the metatibia – apex of metatibia/apex of first metatarsomere/apex of second metatarsomere. Issidae classification follows Wang et al. (2016)'s phylogeny, adapted in Bourgoin et al. (2020).

All the type specimens of the species described below are deposited in the Museum national d’Histoire naturelle (MNHN), Paris, France or in the China West Normal University (CWNU), Nanchong, Sichuan Province, China.

For genitalia study, the abdomen was separated from the specimen using micro-scissors, and then boiled in a 10% NaOH solution for several minutes until the muscles were completely dissolved leaving only tegumentary structures. Final dissection and observation of the abdomen was done in glycerin after rinsing in distilled water several times. Genitalia were finally stored in genitalia vials under the corresponding specimen. Photographs of external morphology and genitalia characters were taken using a Leica DFC camera attached to a Leica M205FA stereomicroscope and further refined with the software LAS X.

Total genomic DNA was extracted from the fore or middle leg from a paratype of *Pusulissus phiaoacensis* sp. nov. and the holotype of *Pusulissus coronomensis* sp. nov. using a Sangon Ezup column animal genomic DNA purification kit. The DNA of the genes (18S rRNA, 28S rRNA, COXI, Cytb) was amplified using the same primers and amplification procedures as in Wang et al. (2016). DNA sequencing was conducted by the Sangon Company (Shanghai, China). Contigs assembly was made using the software Seqman from package DNAstar v5.01 (www.dnastar.com). All sequences obtained in this study were registered in GenBank with accession numbers mentioned below.

MEGA v7.0 (Kumar et al. 2016) was used for performing alignments for a subset of Parahiraciini taxa already analysed in Wang et al. (2016) plus the specimen of *Rhombissus* sp. analysed in Zhao et al. (2019). Four species (two species in Sarimini and two species in Hemisphaeriini) were chosen as outgroups for the analysis. The related taxon name, collecting location, and GenBank accession numbers are in Table 1. Phylogenetic analysis was performed using the software MrBayes v.3.2.4 (Ronquist et al. 2012) using the same method as Wang et al. (2016) except for running with 100 millions generations, sampling every 1000 generations. FIGTREE v1.1.2 (Rambaut 2016) was used to visualize the tree.

**Table 1.** Taxa sampling, collecting locations and GenBank accession numbers used for the phylogenetic study.

Species name	Collecting location	Gene 18S	Gene 28S (D3–D5)	Gene 28S (D6–D7)	COXI	Cytb
*Fortunia* sp.	China, Yunnan, Xishuangbanna, Mengla, Menglun, 21°24'398"N, 101°16'754"E, 705±21 m	KX761487	KX761527	KX761518	KX761498	KX761509
*Flavina hainana* (Wang & Wang, 1999)	China, Hainan, Jianfengling	KX702824	KX761453	MN381846		KX702912
*Gelastyrella litaoensis* Yang, 1994	China, Hainan, Bawangling	KX702823	KX761452	KX702811	KX761461	KX702911
*Macrodarumoides petalinus* Che, Zhang & Wang, 2012	China, Guangxi, Baise, Jinzhongshan, Songshuping	KX702827		KX702856	KX702926	KX702880
*Neodurium hamatum* Wang & Wang, 2011	China, Yunnan, Xishuangbanna, Mengla, Menglun, 21°24'398"N, 101°16'754"E, 705±21 m	KX702818	KX761446	MN381844	KX702920	
*Tetricodissus pandlineus* Wang, Bourgoin & Zhang, 2015	China, Yunnan, Xishuangbanna, Mengla, Nanshahe, 21°36'12.1"N, 101°34'23.9"E, 826±43 m	KX702817	KX761445	KX702807		KX702907
*Tetricodes songae* Zhang & Chen, 2009	China, Guizhou, Kuankuoshui, 1500 m	KX702841	KX761457		KX702925	KX702916
*Pusulissus* sp.	Vietnam, Vinh Phuc, Tam Dao, 21°26'47"N, 105°38'38"E, 748 m	KX761479		KX761485	KX761471	KX761475
*Pusulissus coronomensis* sp. nov.	China, Guangxi, Hezhou, Qichong, 24°13'6"N, 110°48'34"E, 180 m	MT772139	MT772137	MT772136	MT774094	MT774093
*Pusulissus phiaoacensis* sp. nov.	Vietnam, Phia Oac, 1050 m	MW201961			MT774095	MT774092
*Rhombissus* sp.	China, Shaanxi, Zhouzhi, Houzhenzi, 1050 m	MN381855	MN381852	MN381850		MN332231
*Duplexissus punctatulus* Wang, Zhang & Bourgoin, 2019	China, Yunnan, Xishuangbanna, Mengla, Menglun	KX761490	KX761531	KX761520	KX761501	KX761512
*Hemisphaerius lysanias* Fennah, 1978	Vietnam, Khanh Hoa Province, Hon-Ba massif, 12°13'20"N, 109°06'00"E	KX702833	KX761404	KX702860	KX702933	KX702883
*Hemisphaerius coccinelloides* (Burmeister, 1834)	Philippines, Los Banos, UP Hortarium, 14°09'53"N, 121°14'14"E	KX702834	KX761405	KX702861	KX702934	KX702884
*Sarima bifurca* Meng & Wang, 2016	China, Yunnan, Xishuangbanna, Mengla	KX702819	KX761447	KX702808	KX702921	KX761552

## Taxonomy

**Family Issidae Spinola, 1839**

**Subfamily Hemisphaeriinae Melichar, 1906 (sec. Wang et al. 2016)**

### Tribe Parahiraciini Cheng & Yang, 1991

#### Pusulissus gen. nov.

Taxon classificationAnimaliaHemipteraIssidae

480DF2A8-AC73-5B53-AD77-5982056650C9

http://zoobank.org/CBD98E0A-F175-45B4-8199-D57E7B1243A4

Figs 2–7 , 8–12 , 13–14 , 15–20 , 21–24 , 25–30 , 31–35 , 36–37 , 38–44

##### Type species.

*Pusulissus phiaoacensis* sp. nov.

##### Diagnosis.

**Figures 2–7.**
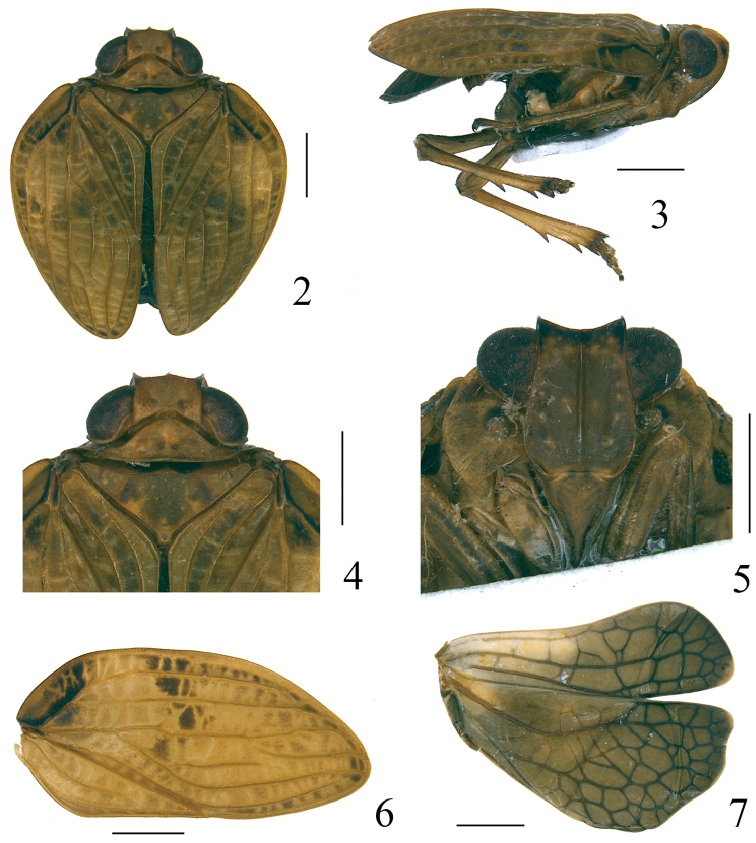
*Pusulissus phiaoacensis* sp. nov. **2** adult (holotype), dorsal view **3** adult (holotype), lateral view **4** head and thorax (holotype), dorsal view **5** adult (holotype), frontal view **6** forewing (paratype) **7** hindwing (paratype). Scale bars: 1 mm.

This genus is similar to *Tetricodes* Fennah, 1956, from which it differs as follows: 1) The absence of the frontal black median tubercle on the disc of frons (Figs 5, 28); 2) Median carina of frons well distinct, extending from the dorsal margin almost to the frontoclypeal suture (Figs 5, 28), while it is only present in the dorsal part of the frons or invisible in *Tetricodes* (Zhang and Chen 2009, fig. 21); 3) The forewing distinctly broadest in the basal 1/3 (Figs 6, 29), while regularly convex in *Tetricodes* (Zhang and Chen 2009, fig. 4).

This genus is also very similar to *Thabena* Stål, 1866, but differs by 1) Its vertex with anterior margin in dorsal view very slightly angularly convex (Fig. 4) or straight (Fig. 27), while roundly convex in *Thabena* (Chen et al. 2014, figs 2–74C); 2) Frons much longer, more than 1.2 times longer in midline than widest part (Figs 5, 28), but wider than long in *Thabena*, less than 0.9 times longer in midline than widest part (Chen et al. 2014, fig. 2–74E).

##### Description.

Head with the compound eyes a little wider than pronotum (Figs 4, 27). Vertex nearly rectangular, slightly wider than long, without median carina or median carina very weakly present; anterior margin very slightly protruded (Fig. 4) or almost straight (Fig. 27), lateral margins parallel, posterior margin angularly concave medially (Figs 4, 27). Frons obviously longer than wide, apical and lateral margins carinated and elevated, dorsal margin deeply concave at middle, frons slightly narrower in the middle level of the compound eyes, then gradually broadened below the lower margin of the compound eyes (Figs 5, 28); median carina obviously elevated from the dorsal margin extending to near base, but not reaching to frontoclypeal suture (Figs 5, 28); disc with several tubercles in its dorsal part and lateral areas (Figs 5, 28). Frontoclypeal suture straight (Fig. 5) or slightly convex (Fig. 28). Clypeus flattened, without median carina or median carina very weak, almost invisible (Figs 5, 28). Rostrum long, reaching to hind coxae; third segment almost as long as second one. Genae in lateral view flattened and oblique, with a small protuberance near base (Figs 3, 26). Pronotum triangular, anterior and posterior margins elevated, without carina (Figs 4, 27), paranotal lobes developed. Mesonotum inverted triangular, a little longer than pronotum at midline, without carina (Figs 4, 27). Forewings twice longer than broad, longitudinal veins obvious and elevated, transverse veins not elevated, but existing in the whole forewing, apical margin oblique, triangular-shaped (Figs 6, 29). Vein ScP+R firstly separated near base after a short common stem, ScP+RA long, reaching apical 1/5 of costal margin, terminal of RP vein reaching to apical margin of forewing (Figs 6, 29); MP vein firstly forked near basal 1/4, MP_1+2_ forked again at apical 1/4, MP_3+4_ forked again at middle, MP_3_ short, just reaching bifurcation of MP_1_ and MP_2_ or slightly beyond bifurcation, MP_4_ long, extending to apical margin of forewing; CuA simple, sinuate, extending to apex of forewing (Figs 6, 29). Clavus closed, reaching to almost middle of forewing, Pcu and A1 fused at apical 1/3 of clavus (Figs 6, 29). Hindwing with longitudinal veins well developed, with a set of numerous transverse veins, CuP-Pcu-A1 lobe distinctly wider than ScP-R-MP-Cu lobe, A2 lobe very narrow with A2 vein absent (Figs 7, 30); MP and CuA not fused, Pcu and A1 also separated (Figs 7, 30). Hind tibia with 2 lateral spines on apical half (Fig. 3). Metatibiotarsal formula: 2-(7-8)/(5-8)/2.

**Figures 8–12.**
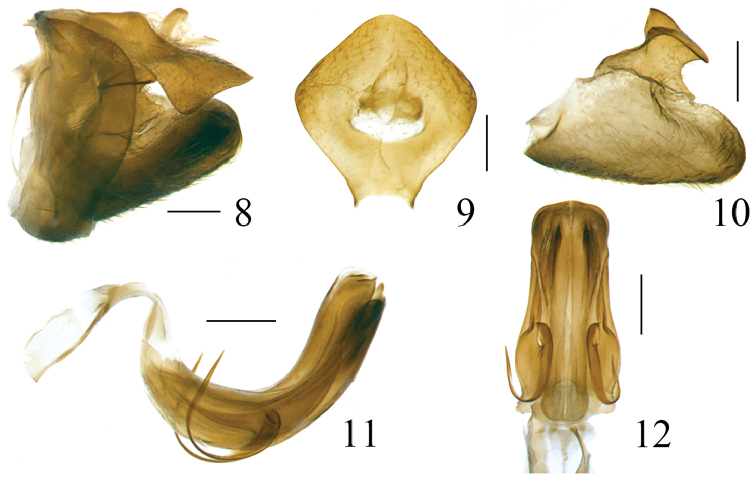
*Pusulissus phiaoacensis* sp. nov., male, holotype. **8** genitalia, lateral view **9** anal tube, dorsal view **10** gonostylus, lateral view **11** phallic complex, right lateral view **12** phallic complex, ventral view. Scale bars: 0.2 mm.

**Figures 13–14.**
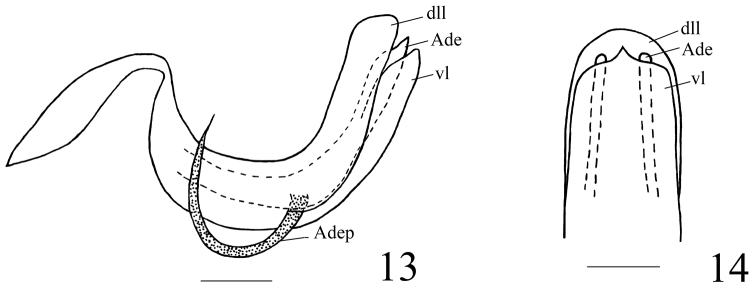
*Pusulissus phiaoacensis* sp. nov., male, holotype. **13** phallic complex, right lateral view **14** apex of phallic complex, posterior view. Abbreviations: dll: dorso-lateral lobe of periandrium; Ade: Aedeagus; vl: ventral lobe of periandrium; Adep: Aedeagus processes. Scale bars: 0.2 mm.

***Male genitalia***. Anal tube in lateral view relatively large and thick, with lateral lobes on apical half extended downwards (Figs 8, 31). Pygofer subrectangular in lateral view (Figs 8, 31). Gonostyli nearly triangular in profile, dorsal margin sinuate, posterior margin deeply concave to a groove in apical half then strongly convex posteriorly, caudo-ventral angle strongly convex and rounded (Figs 10, 33). Capitulum broader than high, triangular, with a relatively long meniscate lateral process with both apical parts obtuse, apical process sharp (Figs 10, 33). Periandrium symmetrical, with dorsal and ventral margins parallel, shallowly U-shaped, divided into dorso-lateral lobe (dll) and ventral lobe (vl), ventral lobe very slightly shorter than dorso-lateral one (Figs 11, 13, 34, 36). Aedeagus (Ade) with a pair of lateral processes (Adep) originating from the middle, directed anteriorly and upcurved (Figs 11, 13, 34, 36).

***Female genitalia*.** Gonoplacs in lateral view nearly rectangular, dorsal margin straight, anterior and posterior margins nearly parallel to each to other, posterior margin with apical half membranous, ventral margin with the needle-shaped basal part (Figs 16, 40); in dorsal view lateral margins convex outward, median area fused in apical half (Fig. 39). Gonapophysis IX in dorsal view widest a little beyond middle then gradually sharpening to apex (Figs 17, 41); in lateral view broad, widest near middle, divided into upper and lower parts, both with sharp tips, bifurcate near apex (Figs 18, 42); upper dorsal margin sinuate, flattened at basal 1/3 and middle part obviously convex upward then gradually sloping posteriorly (Figs 18, 42); ventral lower margin with apical half sloping downwards (Figs 18, 42). Gonospiculum bridge small (Figs 17, 18, 41, 42). Anterior connective lamina of gonapophysis VIII with obscure teeth on apex and outer-lateral margin, inner-lateral margins without teeth (Figs 20, 44). Endogonocoxal process membranous, slightly shorter than anterior connective lamina of gonapophysis VIII (Figs 20, 44). Gonocoxa VIII connects with gonapophysis VIII by a rectangular shape (Figs 20, 44).

**Figures 15–20.**
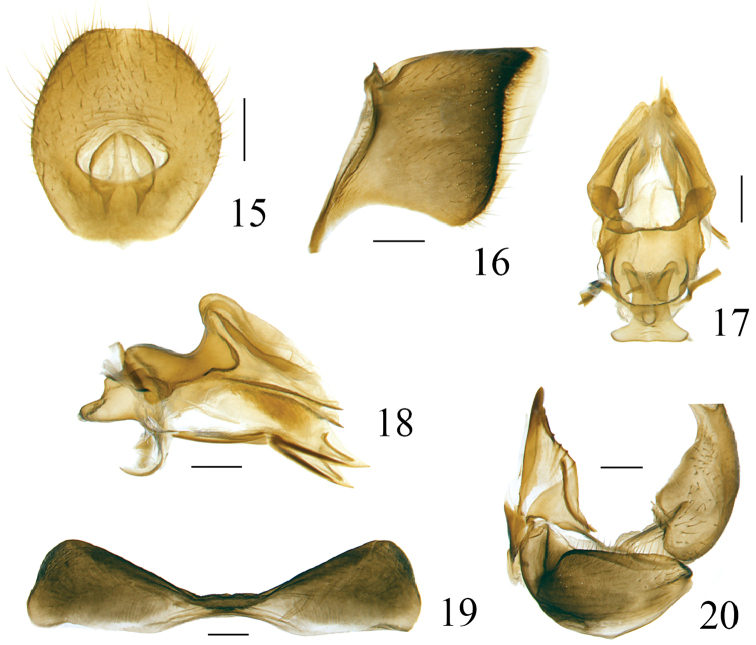
*Pusulissus phiaoacensis* sp. nov., female, paratype. **15** anal tube, dorsal view **16** gonoplac, lateral view **17** gonapophysis IX and gonospiculum bridge, dorsal view **18** gonapophysis IX and gonospiculum bridge, lateral view **19** sternite VII, ventral view **20** gonocoxa VIII and gonapophysis VIII, lateral view. Scale bars: 0.2 mm.

##### Etymology.

The name is an arbitrary association from the Latin word “pusulosus” which means ‘pustulous’, referring to the tubercles or pustules present on the frons and “issus” referring to the family. The name is treated as masculine.

**Figures 21–24.**
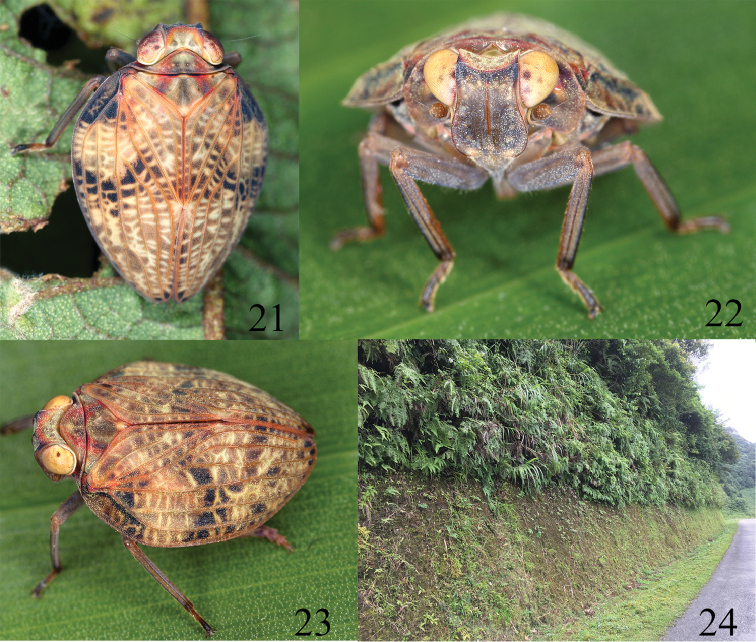
Habitus and habitat of *Pusulissus phiaoacensis* sp. nov. **21–23** habitus in nature (Photo G. Kunz) **24** habitat (Photo T. Bourgoin).

#### Pusulissus phiaoacensis sp. nov.

Taxon classificationAnimaliaHemipteraIssidae

20434C73-CB6B-5346-A9E6-B3A2CCC5E65F

http://zoobank.org/8C827F6F-344C-419A-A1B1-CCFA648CF74B

Figs 2–7 , 8–12 , 13–14 , 15–20 , 21–24

##### Type materials.

***Holotype***: ♂, VIetnam: Phia Oac, 12 vii 2019, 1050 m, (22°26'0.78"N, 105°53'4.692"E), CAV [chasse à vue], rec. Th. Bourgoin, MNHN(EH) 24743. ***Paratypes***: 1♀, same data as holotype, MNHN(EH) 24744 [sequenced specimen]; 1♀, same location and collection date as holotype, but rec. G. Kunz MNHN(EH) 24745.

##### Description.

Length: male (including forewings) (*N* = 1): 5.0 mm; female (including forewings) (*N* = 2): 5.1–5.3 mm.

***Coloration***. For the dry specimens, general coloration tawny (Fig. 2). Vertex tawny, with two brown circular markings near base of disc (Fig. 4). Compound eyes black, supported by tawny callus (Figs 4, 5). Frons brown, dorsal part black (Fig. 5); dorsal and lateral margins carinated and brown, median carina brown (Fig. 5). Frons with two tawny round markings in middle area, dorsal and lateral areas distributed with around ten tawny tubercles on each side (Fig. 5). Antennae brown (Fig. 5). Postclypeus brown (Fig. 5). Genae tawny (Fig. 3). Pronotum tawny, median area with two small brownish impressions on disc (Fig. 4), paranotal lobes tawny (Fig. 5). Mesonotum tawny, with two brown longitudinal bands on disc (Fig. 4). Forewings tawny with veins tawny, basal part of costal area with some irregular black markings, area from basal 1/3 of costal margin extending to middle of forewing surface also with some irregular black markings, apical margin with black linear marking (Fig. 6); some specimens with these black markings almost invisible, but with one extremely large whitish round marking at basal 1/3 of forewing on each side (Fig. 2). Hindwings brown, veins clearer (Fig. 7). Legs tawny (Figs 3, 5). For alive and fresh specimens, vertex, pronotum, mesonotum and forewings interspersed with some reddish markings or coloration as mentioned above (Figs 21–23). Compound eyes yellow (Figs 21–23). Frons brown, dorsal part black, median carina brown, disc distributed with several yellow tubercles (Fig. 22). Legs brown (Figs 21–23).

***Head and thorax***. Vertex 2.1 times wider than long in midline, anterior margin very slightly angularly convex (Fig. 4). Frons 1.3 times longer in midline than widest part, 1.3 times wider at widest part than dorsal margin (Fig. 5). Pronotum 1.4 times longer in midline than vertex in midline (Fig. 4). Mesonotum 2.3 times wider along anterior margin than long in midline (Fig. 4). Forewings 2.1 times longer at longest part than widest part (Fig. 6). Hindwing with apical margin of CuP-Pcu-A1 lobe sinuate (Fig. 7). Metatibiotarsal formula: 2–8/5/2.

***Male genitalia***. Anal tube in lateral view with lateral lobes well visible (Fig. 8); in dorsal view mushroom shaped, widest at middle, as long in midline as widest part, apical margin angularly rounded, lateral margins strongly angularly rounded (Fig. 9); anal opening located near middle of anal tube (Fig. 9). Pygofer in lateral view 2.5 times higher than wide, dorsal margin obviously sloping to the posterior, dorso-lateral angle rounded, posterior margin strongly roundly convex (Fig. 8). Gonostylus in lateral view with dorsal margin slightly sinuate (Fig. 10). Capitulum of gonostylus derived from middle of gonostylus, broad, sharp triangular, directed to anterior; meniscate processes with one side not reaching to anterior margin of capitulum and another side beyond posterior margin of capitulum (Fig. 10). Periandrium in lateral view with dorso-lateral lobe rounded apically (Figs 11, 13). In posterior view, ventral lobe of periandrium with apical margin mostly straight but spinous protruded at middle (Fig. 14). Lateral processes of aedeagus long and slender, hooked, derived from the middle, directed anteriorly to basal 1/4, then curved upward, surpassing the dorsal margin of periandrium (Figs 11, 13); in ventral view this pair of processes curved outwards (Fig. 12).

***Female genitalia***. Anal tube in dorsal view broadly ovate, 1.1 times longer in midline than widest part, widest at middle, apical margin nearly straight, lateral margins rounded, anal opening situated slightly below middle (Fig. 15). Anterior connective lamina of gonapophysis VIII with two obscure large teeth in the apex and four small keeled teeth in outer-lateral group (Fig. 20). Gonocoxa VIII subquadrangular (Fig. 20). Hind margin of sternite VII roundly concave, with the median part nearly straight (Fig. 19).

##### Etymology.

The name refers to the locality where the new species was found.

##### Habitat.

The species was swept from pteridophytes at the margin of the mountainous rainforest (around 1050 m altitude) beside a road (Fig. 24).

##### Note.

Genes sequences were registered in GenBank with the following accession numbers: MW201961 (18S), MT774095 (COXI), MT774092 (Cytb). *P. phiaoacensis* differs from another unnamed *Pusulissus* species (referred here as *Pusulissus* sp.) (Fig. 46) by 25 bp in the length 681 bp of COXI. Both species are from Vietnam.

#### Pusulissus coronomensis sp. nov.

Taxon classificationAnimaliaHemipteraIssidae

34D67C1B-ACCA-51C7-B40A-C80738F83558

http://zoobank.org/7537121D-C88F-44EC-8E7E-47696162D02A

Figs 25–30 , 31–35 , 36–37 , 38–44

##### Type materials.

***Holotype***: ♂, China: Guangxi Province, Hezhou, Qichong natural reserve, 24°13'6"N, 110°48'34"E, 180 m, 7 viii 2018, coll. Feilong Yang & Kun Zhao (CWNU) [sequenced specimen]. ***Paratypes***: 1♂, 1♀, same data as holotype (CWNU).

##### Differential diagnosis.

This new species is very similar to *P. phiaoacensis* from Vietnam, but differs as follows: 1) Apical margin of the Pcu-A1 lobe on hindwing round (Fig. 30), while in *P. phiaoacensis* it is sinuate (Fig. 7); 2) Pair of lateral processes on aedeagus shorter, reaching to the ventral margin of periandrium (Fig. 34), while in *P. phiaoacensis* they surpass the dorsal margin of periandrium (Fig. 11); 3) Dorsal margin of female anal tube concave at middle (Fig. 38), while in *P. phiaoacensis* it is nearly straight (Fig. 15).

##### Description.

Length: male (including forewings) (*N* = 2): 5.1–5.3 mm; female (including forewings) (*N* = 1): 5.3 mm.

**Figures 25–30.**
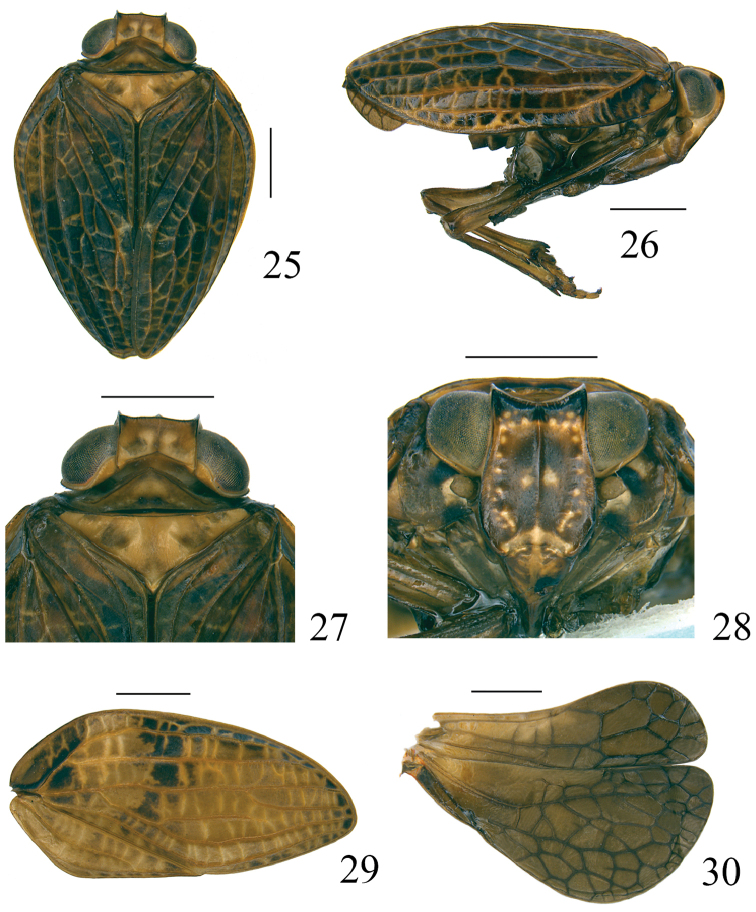
*Pusulissus coronomensis* sp. nov. **25** adult (holotype), dorsal view **26** adult (holotype), lateral view **27** head and thorax (holotype), dorsal view **28** adult (holotype), frontal view **29** forewing (paratype) **30** hindwing (paratype). Scale bars: 1 mm.

***Coloration***. General appearance brown (Figs 25, 26). Vertex tawny, with two brown circular markings near the base, midline brown, margins black (Fig. 27). Compound eyes brownish dark, supported by tawny callus (Fig. 27). Frons brown, dorsal part black (Fig. 28); dorsal and lateral margins carinated by black, median carina brown (Fig. 28). Frons with two yellowish round markings in middle area, apical and lateral areas distributed with around twelve yellowish tubercles on each side, basal part yellow (Fig. 28). Antennae brown (Fig. 28). Postclypeus brown mixed with some tawny (Fig. 28). Genae tawny (Fig. 26). Pronotum tawny, median area with two brownish small impressions (Fig. 27), paranotal lobes brown mix with some yellow (Fig. 28). Mesonotum tawny (Fig. 27). Forewings tawny with veins tawny, the basal part of costal area with some irregular black markings, the middle area from basal third of costal margin extending to the middle of forewing surface also have some irregular black markings, the apical margin with a black linear marking (Fig. 29); some specimens with the black markings on forewing almost invisible, but with an obscure large paler round marking at basal third on each side of forewing (Fig. 25). Hindwings brown (Fig. 30). Legs tawny (Fig. 26).

***Head and thorax*.** Vertex 2.3 times wider than long in midline, anterior margin straight, posterior margin angularly concave at middle, but the level shallower than the new species described above (Fig. 27). Frons 1.2 times longer in midline than the widest part, 1.2 times wider at widest part than dorsal margin (Fig. 28). Pronotum 1.1 times longer in midline than vertex in midline, lateral margins straight and oblique (Fig. 27). Mesonotum 2.2 times wider along anterior margin than long in midline (Fig. 27). Forewings twice longer at longest part than widest part (Fig. 29). Hindwing with apical margin of CuP-Pcu-A1 lobe rounded (Fig. 30). Metatibiotarsal formula: 2-(7–8)/(6–8)/2.

**Figures 31–35.**
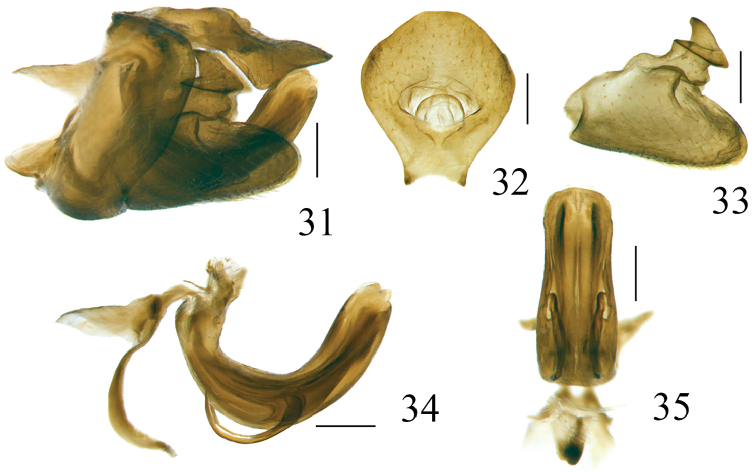
*Pusulissus coronomensis* sp. nov., male, holotype. **31** genitalia, lateral view **32** anal tube, dorsal view **33** gonostylus, lateral view **34** phallic complex, right lateral view **35** phallic complex, ventral view. Scale bars: 0.2 mm.

**Figures 36–37.**
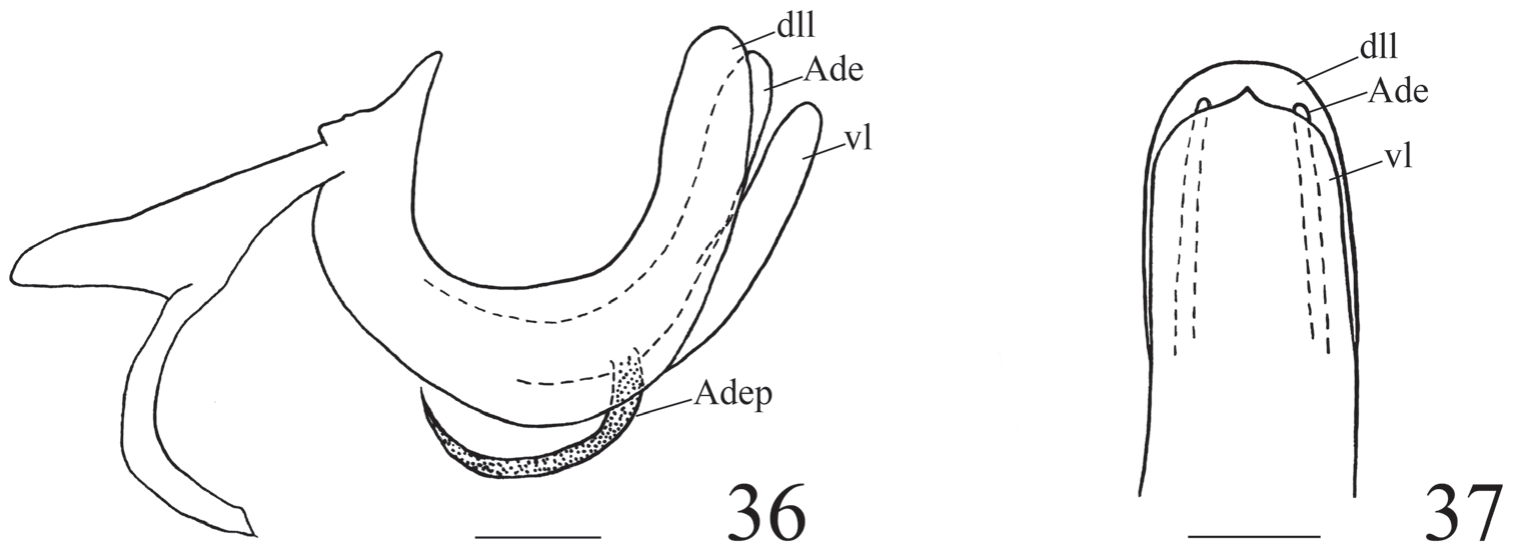
*Pusulissus coronomensis* sp. nov., male, holotype. **36** phallic complex, right lateral view **37** apex of phallic complex, posterior view. Abbreviations: dll: dorso-lateral lobe of periandrium; Ade: Aedeagus; vl: ventral lobe of periandrium; Adep: Aedeagus processes. Scale bars: 0.2 mm.

***Male genitalia*.** Anal tube in dorsal view mushroom shaped, widest beyond middle, as long in midline as widest part, apical margin rounded (Fig. 32), lateral margins roundly convex in dorsal view (Fig. 32) and visible in lateral view (Fig. 31); anal opening located near the middle of anal tube (Fig. 32). Pygofer in lateral view subrectangular, dorsal margin slightly sloping to the posterior, dorso-lateral angle rounded, posterior margin almost parallel with the anterior margin (Fig. 31). Gonostylus in lateral view with dorsal margin elevated at the basal third (Fig. 33). Capitulum of gonostylus derived after the middle of gonostylus, broad, sharp triangular, directed to anterior; the meniscate processes with one side beyond the base of anterior margin of capitulum and another side beyond the posterior margin of capitulum (Fig. 33). In lateral view, periandrium dorso-lateral lobe and ventral lobe rounded apically (Figs 34, 36). In posterior view, ventral lobe of periandrium spinous protruded at middle in apical margin (Fig. 37). The lateral processes of aedeagus relatively short, hook-shaped, derived from the middle, directed anteriorly to basal fourth, reaching the ventral margin of periandrium (Figs 34, 36); in ventral view this pair of processes directed downwards (Fig. 35).

**Figures 38–44.**
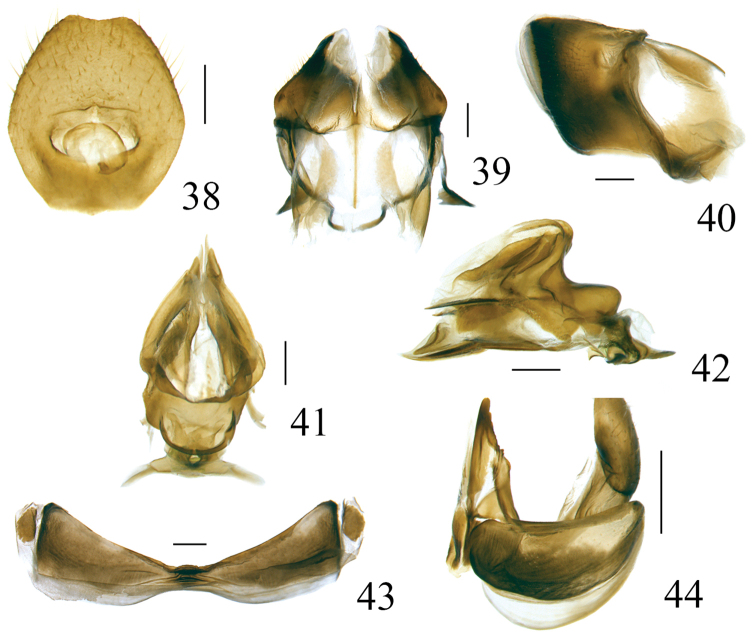
*Pusulissus coronomensis* sp. nov., female, paratype. **38** anal tube, dorsal view **39** gonoplacs, ventral view **40** gonoplacs, lateral view **41** gonapophysis IX and gonospiculum bridge, dorsal view **42** gonapophysis IX and gonospiculum bridge, lateral view **43** sternite VII, ventral view **44** gonocoxa VIII and gonapophysis VIII, lateral view. Scale bars: 0.2 mm.

***Female genitalia*.** Anal tube in dorsal view ovate, 1.1 times longer in midline than widest part, widest before mid length, apical margin slightly concave at middle, lateral margins rounded, anal opening situated slightly below middle (Fig. 38). Anterior connective lamina of gonapophysis VIII with two obscure large teeth in the apex but the keeled teeth in outer-lateral margin invisible (Fig. 44). Gonocoxa VIII long, quadrangular (Fig. 44). Hind margin of sternite VII roundly concave, the median part with a small convex protuberance (Fig. 43).

##### Etymology.

Arbitrary euphonic name referring to the crown (latin ‘corona’) of yellow pustules on the frons.

##### Note.

Genes sequences were registered in GenBank with the following accession numbers: MT772139 (whole 18S), MT772137 (28S D3–D5), MT772136 (28S D6–D7), MT774094 (COXI) and MT774093 (Cytb). For COXI of 681 bp length, this species differs by 57 bp with *P. phiaoacensis* and by 50 bp with *Pusulissus* sp. (Fig. 46).

#### *Pusulissus* gen. nov. distribution

**Figure 45.**
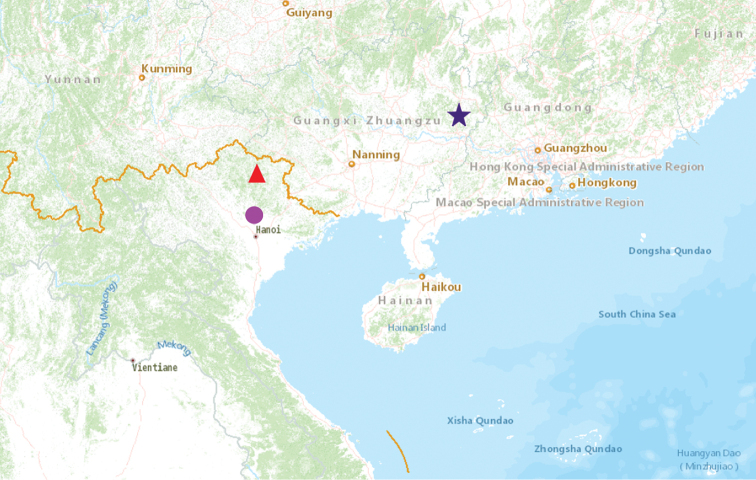
Distribution map of *Pusulissus*. The red triangular, blue five-pointed star and purple circular respectively indicate the distribution of species *P. phiaoacensis* sp. nov., *P. coronomensis* sp. nov. and *Pusulissus* sp.

With three different species, the new genus *Pusulissus* appears distributed around the South China in the Guangxi Province (Hezhou, Qichong Natural Reserve) and in North Vietnam (Cao Bang Province: Phia Oac and Vinh Phuc Province: Tam Dao) (Fig. 45). The Vietnamese specimens were collected in mountainous biotopes at relatively high altitudes (between 750 and 1050 m) while at lower altitude (180 m) in China.

#### *Pusulissus* gen. nov. phylogeny

**Figure 46.**
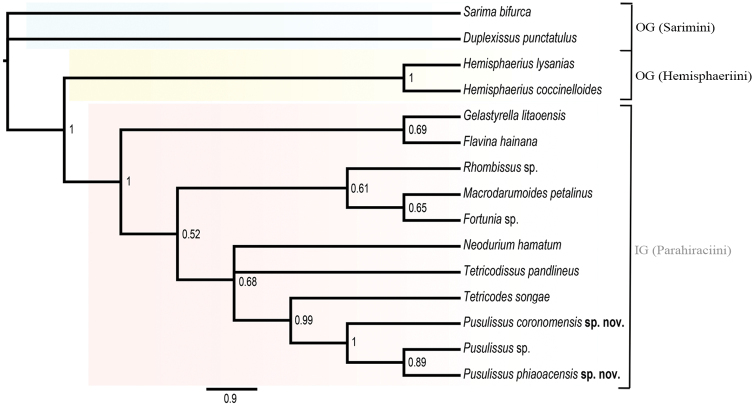
Bayesian 50% consensus tree of Parahiraciini species based on combined sequences (18S, 28S, COXI, Cytb) with 2 species of Sarimini and 2 species of Hemisphaeriini as outgroup. At each node, values denote posterior probability support.

The new genus *Pusulissus* refers to the taxon “Gen. nov. *apud Tetricodes*” in Wang et al. (2016)’s molecular phylogenetic analyses, and three different species are observed in this study (Fig. 46). However, only two species are formally described here as the third taxon, *Pusulissus* sp., corresponding to the one already sequenced as “Gen. nov. *apud Tetricodes*” in Wang et al. (2016), is represented by only one incomplete female specimen from Vietnam also: Tam Dao, Vinh Phuc Province.

Within Parahiraciini, and from the combined genes (18S rRNA, 28S rRNA, COXI and Cytb) phylogeny (Fig. 46), the new genus *Pusulissus* appears sister to the genus *Tetricodes* Fennah, 1956, and both are sister to the genus *Tetricodissus* Wang, Bourgoin & Zhang, 2015 and *Neodurium* Fennah, 1956 in this study (Fig. 46), while this topology was ((*Pusulissus + Tetricodes*) + (*Tetricodissus + Neodurium*)) in Wang et al. (2016). *Pusulissus phiaoacensis* sp. nov., *Pusulissus* sp. and *Pusulissus coronomensis* sp. nov. respectively differ by 98 bp, 89 bp and 101 bp with the species *Tetricodes songae* Zhang & Chen, 2009 on a total of 681 bp in COXI.

#### Regard to the tribe Parahiraciini

The Parahiraciini lineage was erected within the Issidae by Cheng and Yang (1991a) for the single genus *Parahiracia* Ôuchi, 1940 with a subfamily rank. The subfamily recognition was based on the elongate ovate body, the absence of wax plates of abdominal segment VII-VIII (present in other Issidae sec. Fennah 1954) and the presence of respectively 10 and 8 median sensory pits on each side of the meso- and metanotum in the 5^th^ instar larva, versus 2–5 and 0–2 sensory pits in other issids (Cheng and Yang 1991a, b). This lineage was confirmed as a monophyletic group by several successive molecular analyses (Wang et al. 2016; Bourgoin et al. 2018, unpublished data) while it was not recovered by Gnezdilov et al. (2020), probably due to methodological biases which is out of the scope of this paper.

From a morphological perspective and for the adults, Gnezdilov and Wilson (2007) characterized the tribe by 1) beetle-like conformation, convex, elongate, and apically narrowed forewings not exceeding the length of the abdomen with a net of apical transverse veins and 2) long fore and middle legs. Gnezdilov and Wilson (2007) added a “well-developed three- or two lobed (anal lobe more or less reduced) hindwings with a deep notch between remigium and vannus and a net of transversal veins in the distal part”, but regarded this last character as plesiomorphic. In their review and identification key to genera, Zhang and Chen (2012, 2013) used characters of the head capsule (presence or absence of swollen frons, vertex conformation), prothoracic femora and tibiae (flattened or not), forewing conformation and claval suture (present or not) and some genitalia characters. However, none of these characters appear as specific of the tribe. More recently, Gnezdilov (2017) added the “cuspidal apex of clavus” (Gnezdilov 2015) in the diagnosis of the tribe and also retained (Gnezdilov 2018) the narrow anal lobe, the deep cubital cleft, and the cuspidal apex of the forewing clavus as apomorphies of the tribe.

**Figures 47–52.**
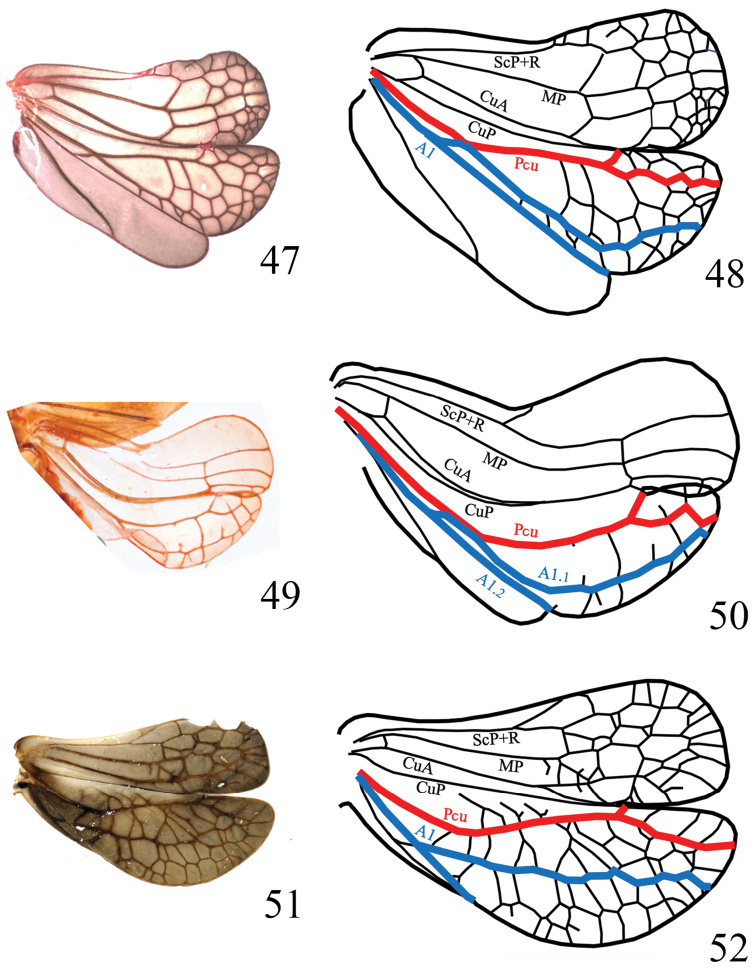
Hindwing pattern and venation. **47, 48**Scantiniina subtrib. nov.: *Scantinius bruchoides* (Walker, 1858) (modified from Gnezdilov 2012, fig. 3) **49, 50**Vindilisina subtrib. nov.: *Vindilis fornicata* Stål, 1870 (modified from Gnezdilov 2018, fig. 3) **51, 52**Parahiraciina Cheng & Yang, 1991 subtrib. nov.: *Tetricodes tamdaoensis* Vanslembrouck & Constant, 2018 (modified from Vanslembrouck and Constant 2018, fig. 4).

In 2016, Wang et al. (2016) proposed a new classification of the family, confirming Cheng and Yang (1991a)’s separation of Parahiraciini with the recognition of several other new tribes. They put in light the importance of the hindwing conformation in adults that appeared being well characteristic for each lineage. Since, our further studies of many Oriental issid genera have confirmed this view and a clearer figure for Parahiraciini taxa (including several yet non-described new genera) has now emerged. We can now provide a non-ambiguous morphological definition of the tribe Parahiraciini allowing us to review its composition and to divide the tribe into three subtribes: Scantiniina subtribe nov., Vindilisina subtribe nov., and the nominal one Parahiraciina Cheng & Yang, 1991 subtribe nov., although we don’t exclude that the first two might represent in the future distinct lineages of higher range value. The following subtribal diagnoses are therefore proposed, mainly based on the hindwing conformation (Figs 47–52), with a key to Parahiraciini subtribes.

##### Parahiraciini

Taxon classificationAnimaliaHemipteraTropiduchidae

Cheng & Yang, 1991

B660DC42-3D26-54D8-916C-8694F918D1EE

Type genus: Parahiracia Ôuchi, 1940 [syn. of Fortunia Distant, 1909 (Gnezdilov et al. 2004)]

###### Diagnosis

(modified from Gnezdilov 2017, 2018). Body more or less flattened dorso-ventrally, frons often projecting, usually with a proboscis, forewings usually with keel-shaped veins, caudo-dorsal angle of clavus of forewings usually in shape of distinct triangular lobe (cuspidal apex of clavus: Gnezdilov 2015). Hindwing 2 or 3-lobate, strongly notched at CuP with CuP-Pcu-A1 lobe generally slightly wider than Sc-R-MP-CuA lobe; the two lobes almost the same length. Pcu and A1_1_ merging or not in basal half of forewing. A2 lobe wide or reduced. Net of transverse veins present in Sc-R-MP-CuA lobe, or absent.

##### Parahiraciina

Taxon classificationAnimaliaHemipteraTropiduchidae

Cheng &Yang, 1991 subtribe nov.

66209CDB-3B6A-5AAC-B70C-39FCC8A9254B

http://zoobank.org/84516219-0226-4258-BED7-A476E54DA37F

Figs 51 , 52

###### Type genus.

*Parahiracia* Ôuchi, 1940 [syn. of *Fortunia* Distant, 1909 (Gnezdilov et al. 2004)]

###### Diagnosis.

The subtribe is identified and separated according to the following list of characters:

1. Hindwings bilobate, strongly notched at CuP with CuP-Pcu-A1 lobe generally wider than Sc-R-MP-CuA lobe; the two lobes almost the same length.
2. Posterior margin not or indistinctly notched at A1 2.
3. A2 lobe with anal area posterior to A1 strongly reduced, much shorter and much thinner than the anterior lobes.
4. Sc-R-MP-CuA and CuP-Pcu-A1 lobes covered with a set of numerous transverse veins.
5. CuA and CuP not merging before the anterior notch.
6. Pcu and A1 1 not merging in basal half of forewing.
7. A2 present, not branched or absent. In some species a transverse a2-a1 connecting A2 with A1 at the level of its basal branching (Tetricodes tamdaoensis Vanslembrouck & Constant, 2018).

###### Note.

Based on this diagnosis, Parahiraciina constitutes a well-defined group supported by several apomorphic characters (reduced anal lobe, numerous transverse veins) and the molecular analysis results. Accordingly, genera *Scantinius*, *Vindilis* and *Nisoprincessa* are moved to separate new subtribes. They probably all belong to the same higher lineage Parahiraciini based on the apomorphic strong cubital notch of the hindwing.

While *Folifemurum*, was already excluded from Parahiraciini by Wang et al. (2016), this view was not followed by Gnezdilov (2017). Based on its rounded hemisphaeriini general shape, its apomorphic one lobed hind wing, the medio-carinated frons, and particularly according to the molecular analysis result, it is here transferred to HemisphaeriiniMongolianina. *Gelastyrella* is here maintained as a valid genus following Chen et al. (2014) versus Gnezdilov (2009)’s synonymy with *Thabena*, according to the number of small spines on the first metatarsi (more than 35 in *Gelastyrella* while less than 21 in *Thabena*), the large corpus connective of the phallic complex bearing a large and obvious ventrad expansion (corpus connective reduced and phallic complex without ventrad expansion in *Thabena*), and posterior margin of female sternite VII medially quadrate-shaped (triangular in *Thabena*) (Chen et al. 2014).

##### Scantiniina

Taxon classificationAnimaliaHemipteraIssidae

subtribe nov.

99C2C398-23B4-53C7-9E70-79F28E7EE1E5

http://zoobank.org/D6FB9F06-AAE0-41F7-9DE2-9EB88A950398

Figs 47 , 48

###### Type genus.

*Scantinius* Stål, 1866.

###### Diagnosis.

The subtribe is identified and separated according to the following list of characters:

1. Hindwing trilobate, strongly notched at CuA2 with CuP-Pcu-A1 lobe slightly longer, as wide as Sc-R-MP-CuA lobe.
2. Posterior margin distinctly notched at A1 2.
3. Anal lobe posterior to A1 present, surpassing half-length of CuP-Pcu-A1 lobe, apically rounded and 1/2 thinner than the anterior ones, and with margins sub-parallel.
4. Sc-R-MP-CuA and CuP-Pcu-A1 lobes covered with a set of numerous transverse veins.
5. CuA and CuP merging at the anterior notch.
6. Pcu and A1 merging in basal half of forewing.
7. A2 present, single.

###### Note.

This subtribe is currently monogeneric and distributed in Indonesia (Sumatra), in Peninsular Malaysia and Sarawak (Gnezdilov and Wilson 2007). Based on the strong CuA2-CuP notch and the reticulated Sc-R-MP-CuA and CuP-Pcu-A1 lobes, it is tentatively considered as a sister lineage to Parahiraciina, but without molecular data, its place in the phylogeny of the Hemisphaeriinae sec. Wang et al. (2016) remains uncertain.

##### Vindilisina

Taxon classificationAnimaliaHemipteraIssidae

subtribe nov.

CF5FF936-0841-5C5E-AE1B-E3997C8AA4E1

http://zoobank.org/FFC85FD7-1DD0-448D-9ABC-A064E19A4E30

Figs 49 , 50

###### Type genus.

*Vindilis* Stål, 1870.

###### Diagnosis.

The subtribe is identified and separated according to the following list of characters:

Head capsule with vertex and frons in a slightly convex margin in lateral view. Compound eyes elongated, almost twice as long as wide in lateral view.

1. Hindwing trilobate, strongly notched at CuA2-CuP; Sc-R-MP-CuA and CuP-Pcu-A1 lobes almost the same wide.
2. Posterior margin distinctly notched at A1 2.
3. A2 lobe surpassing half-length of CuP-Pcu-A1 lobe, apically rounded and about 1/4 thinner than the anterior one, and with margins sub-parallel.
4. Sc-R-MP-CuA lobe not covered with a set of numerous transverse veins, a few incomplete ones in CuP-Pcu-A1 lobe.
5. CuA and CuP merging well before the anterior notch.
6. Pcu and A1 merging in basal half of forewing on some distance.
7. A2 present, single.

###### Note.

The genera *Vindilis* and *Nisoprincessa* are transferred in this new subtribe, which is currently distributed only in Philippines (Palawan) (Gnezdilov 2017). The strong CuA-CuP notch looks similar to the other Parahiraciini, but both genera lack the reticulate venation of the Parahiraciina subtribe nov. or Scantiniina subtribe nov. In return, with exception of Hemisphaeriini, they share with Scantiniina subtribe nov. and other Hemisphaeriinae taxa the distinct plesiomorphic trilobate conformation of the hindwing. With Sarimini Wang, Zhang & Bourgoin, 2016 and Kodaianellini Wang, Zhang & Bourgoin, 2016, they exhibit the basal apomorphic merging of veins Pcu and A1. Without molecular data, its position in the phylogeny of Hemisphaeriinae remains uncertain and thus is currently left as a subtribe in the Parahiraciini.

#### Key to Parahiraciini subtribes

**Table:** 

1	Hindwing trilobate, with a distinct notch at A1_2_ vein apex separating a developed anal area apically widely rounded. Pcu and A1_1_ merging in basal half of forewing	**2**
–	Hindwings bilobate, indistinctly notched at A1_2_ apex; anal area short, triangular. Pcu and A1_1_ not merging in basal half of forewing. Sc-R-MP-CuA and CuP-Pcu-A1 lobes covered with a set of numerous transverse veins (Figs 51, 52)	**Parahiraciina Cheng & Yang, 1991 subtribe nov.**
2	CuA and CuP merging at the anterior cubital notch. Sc-R-MP-CuA and CuP-Pcu-A1 lobes covered with set of transverse veins (Figs 47, 48)	**Scantiniina subtribe nov.**
–	CuA and CuP merging well before at the anterior cubital notch. Transverse veins almost absent in Sc-R-MP-CuA lobe (m-cu and r-m present), a few ones often incomplete in CuP-Pcu-A1 lobe (Figs 49, 50). Compound eyes elongated, almost twice as long as wide in lateral view	**Vindilisina subtribe nov.**

## Conclusions

Parahiraciini constitutes a well-defined lineage in IssidaeHemisphaeriinae, easily recognized by the apomorphic cubital strong notch of the hindwing. Its monophyly is also fully supported by the molecular analyses at least for the nine genera, including *Pusulissus* gen. nov., described in this paper (Wang et al. 2016, Bourgoin et al. 2018, this paper). Paraphyly reported by Gnezdilov et al. (2020) in the maximum likelihood tree (fig. 2 pink box) is probably biased, as much as its Bayesian analysis also recovered a 100% supported node for the tribe (fig. 1). The three new subtribes proposed here are easily separated on morphological characters based on the hindwing conformation but their exact placement in the classification remains to be confirmed by molecular analysis. Within Parahiraciini, the placement of *Pusulissus* gen. nov. in the phylogeny in respective to the other Parahiraciina genera, remains of course provisional until a wider sampling would be available, but probably is close to the genus *Tetricodes*.

## Supplementary Material

XML Treatment for Pusulissus

XML Treatment for Pusulissus phiaoacensis

XML Treatment for Pusulissus coronomensis

XML Treatment for Parahiraciini

XML Treatment for Parahiraciina

XML Treatment for Scantiniina

XML Treatment for Vindilisina
